# Tumor Necrosis Factor-*α* Promotes the Tumorigenesis, Lymphangiogenesis, and Lymphatic Metastasis in Cervical Cancer via Activating VEGFC-Mediated AKT and ERK Pathways

**DOI:** 10.1155/2023/5679966

**Published:** 2023-04-21

**Authors:** Xiao Chen, Luping Lin, Qiaoling Wu, Sang Li, Huihui Wang, Yang Sun

**Affiliations:** ^1^Department of Gynecology, Clinical Oncology School of Fujian Medical University, Fujian Cancer Hospital, Fuzhou 350000, China; ^2^Department of Abdominal Medical Oncology, Clinical Oncology School of Fujian Medical University, Fujian Cancer Hospital, Fuzhou 350000, China; ^3^Wenzhou Central Hospital, The Second Affiliated Hospital of Shanghai University, China

## Abstract

**Background:**

Lymphatic metastasis is a common phenomenon of cervical cancer. Tumor necrosis factor-*α* (TNF-*α*) was found to be closely associated with lymphatic cancer metastasis. However, the mechanism through which TNF-*α* regulates lymphatic metastasis in cervical cancer remains unclear.

**Methods:**

In this study, cervical cancer cells were cultured in Dulbecco's modified Eagle's medium (DMEM) with or without TNF-*α* for 48 h, and then the corresponding conditional medium (CM-TNF-*α* or CM) was collected. The level of vascular endothelial growth factor (VEGFC) in the corresponding CM was then detected using an enzyme-linked immunosorbent assay (ELISA). Next, human lymphatic endothelial cells (HLECs) were cultured in CM-TNF-*α* or CM for 48 h. Cell viability was measured using the cell counting kit-8 (CCK-8) assay, and angiogenesis was detected using a tube formation assay. Subsequently, the expressions of AKT, p-AKT, ERK, and p-ERK in HLECs were detected using western blotting. In addition, to further investigate the effect of TNF-*α* on the progression of cervical cancer, a C33A subcutaneous xenograft model was established *in vivo*.

**Results:**

We found that TNF-*α* significantly stimulated cervical cancer cells to secrete VEGFC. Additionally, the CM collected from the TNF-*α*-treated cervical cancer cells notably promoted the proliferation, migration, and angiogenesis of HLECs; however, these changes were reversed by MAZ51, a VEGFR3 inhibitor. Moreover, TNF-*α* obviously elevated D2-40 and VEGFC protein expressions in tumor tissues, promoting lymphangiogenesis and lymphatic metastasis *in vivo*. Meanwhile, TNF-*α* markedly upregulated p-AKT and p-ERK expressions in tumor tissues, whereas these changes were reversed by MAZ51.

**Conclusion:**

Collectively, TNF-*α* could promote tumorigenesis, lymphangiogenesis, and lymphatic metastasis *in vitro* and *in vivo* in cervical cancer via activating VEGFC-mediated AKT and ERK pathways. These results may provide new directions for the treatment of cervical cancer.

## 1. Introduction

Cervical cancer remains one of the main cancer in women, particularly in developing countries [1–4]. At present, cervical cancer is the main cause of mortality in women [2, 5, 6]. Lymphatic metastasis has been identified as a risk factor for cervical cancer recurrence [7]. The formation of lymphatic microvessels is the earliest stage of lymphatic metastasis [8]. At present, clinical treatments for cervical cancer include surgical treatment, radiotherapy, and chemotherapy [9–11]. However, the prognosis of patients with advanced cervical cancer remains relatively poor [12].

Tumor microenvironment (TME) refers to the environment around a tumor, including the surrounding blood vessels, immune cells and son on [13]. A prominent feature of the TME is the recruitment of a large number of inflammatory cells and the production of inflammatory factors [14]. Inflammatory factors are a double-edged sword for tumors [15–17]. On the one hand, they can directly kill tumor cells or induce immune cells to recognize tumor antigens [18, 19]. On the other hand, they can promote tumor cell invasion and metastasis [20]. Thus, it is necessary to explore the relationships among inflammatory factors, lymphatic microvessel formation, and tumorigenesis in cervical cancer.

The extensively studied pro-inflammatory cytokine, tumor necrosis factor-*α* (TNF-*α*) [21], can be secreted by various cells such as adipocytes, activated monocytes, macrophages, B cells, and T cells [22, 23]. Reports have suggested that TNF-*α* can promote tumor development and metastasis [24] by inducing epithelial-mesenchymal transition (EMT) as demonstrated by Yoshimatsu et al. inoral squamous cell carcinoma [25]. In addition, Fujiki et al. reported that TNF-*α* was able to facilitate the occurrence and development of gastric cancer [26]. Nevertheless, the relationship among TNF-*α*, lymphatic microvessel formation, and tumorigenesis in cervical cancer remains unclear. The aim of the present study was therefore to investigate the mechanism through which TNF-*α* regulates lymphatic microvessel formation in cervical cancer.

## 2. Materials and Methods

### 2.1. Cell Culture

HeLa and C33A human cervical cancer cell lines were purchased from the American Type Culture Collection and cultured in DMEM (Thermo Fisher Scientific, Inc.) supplemented with 10% fetal bovine serum (FBS, Thermo Fisher Scientific, Inc.) and 1% penicillin/streptomycin (Thermo Fisher Scientific, Inc.) with 5% CO_2_ at 37°C. HLECs were obtained from Procell and cultured in a medium supplemented with 10% FBS, 1% penicillin/streptomycin, and 1% endothelial cell growth supplement (ECGS, CELL RESARCH) [27].

### 2.2. Conditional Medium (CM)

HeLa and C33A cells were stimulated with different concentrations of TNF-*α* (0, 5, and 10 ng/ml) for 48 h at 37°C, and the corresponding CM (CM-TNF-*α*) was collected. Meanwhile, in the control group, cervical cancer cell lines were cultured in DMEM for 48 h at 37°C, and the corresponding CM was collected [28]. The CM was supplemented with 1% ECGS. After that, HLECs were cultured in CM with or without MAZ51 treatment.

### 2.3. ELISA Analysis

The level of VEGFC in the CM was detected using a VEGFC assay kit (cat. no. H046; Nanjing Jiancheng Bioengineering) [29]. The VEGFC inhibitor MAZ51 was purchased from MedChemExpress (cat. no. HY-116624).

### 2.4. CCK-8 Assay

Cell viability was measured using the CCK-8 assay by culturing HLECs in either CM or CM-TNF-*α* for 48 h, followed by incubation with 10 *μ*l CCK-8 reagent (cat. no. C0047; Beyotime) for 2 h, and then the absorbance was measured at 450 nm using a microplate reader (Thermo Fisher Scientific) [30].

### 2.5. 5-Ethynyl-2′-Deoxyuridine (EdU) Staining

Cell proliferation was measured using an EdU staining assay. The EdU detection kit was purchased from Guangzhou RiboBio (cat. no. C10310-1). Firstly, HLECs were cultured in CM or CM-TNF-*α* for 48 h. Next, HLECs were incubated with 100 *μ*l of 50 *μ*m EdU for 1 h, washed with PBS, and incubated with 1 mg/ml DAPI for 10 min. Next, the EdU-positive HLECs were measured using a fluorescence microscope (IX51; Olympus) [31].

### 2.6. Tube Formation Assay

The number of tube node formed in the HLECs was observed using a tube formation assay. Matrigel-coated 24-well Transwell^®^ (8 *μ*m pore size) was purchased from Corning, Inc. HLECs were cultured in CM or CM-TNF-*α* for 48 h. Next, HLECs (1 × 10^5^ cells) were placed in the Matrigel^®^-coated well at 37°C. Next, the number of tube node of HLECs was observed using a microscope (IX51; Olympus) [32].

### 2.7. Wound Healing Assay

Cell migration was determined using the wound-healing assay by culturing HLECs (5 × 10^5^/cell) in a 6-well plate overnight, creating a wound in the monolayer using a 200 *μ*l pipette tip, washing the cells with PBS, and then observing the scratch widths using a microscope (IX51; Olympus) after 0 and 24 h [31].

### 2.8. Transwell Migration Assay

Transwell assay was used by adding HLECs to the upper chamber containing serum-free DMEM andDMEM with 10% FBS t the lower chamber. The cells that had migrated to the lower chamber after24 hours of incubation were stained with crystal violet dye (cat. no. AS1086; ASPEN) and observed using a microscope (IX51; Olympus) [33].

### 2.9. Western Blotting

The total protein from cells and tumor tissues was extracted, the protein concentration was quantified using a BCA Protein Assay Kit (cat. no. AS1086; Aspen Biosciences) and 40 *μ*g per lane of protein was separated by 10% SDS-PAGE and transferred onto PVDF membranes (EMD Millipore). The membranes were then incubated with primary antibodies: anti-AKT (1 : 1,000; cat. no. AF0836), anti-p-AKT (1 : 1,000; cat. no. 28731-1-AP), anti-ERK (1 : 1,000; cat. no. 11257-1-AP), anti-p-ERK (1 : 1,000; cat. no. 28733-1-AP), and anti-GAPDH (1 : 1,000; cat. no. 60004-1-Ig) overnight at 4°C. GAPDH was used as the internal reference. Next, the membranes were incubated with corresponding HRP-conjugated secondary antibodies for 1 h at room temperature. Finally, an enhanced chemiluminescent substrate kit (cat. no. AS1059; Aspen Biosciences) was used to observe the protein bands [34]. Anti-AKT, anti-p-AKT, anti-ERK, anti-p-AKT, and anti-GAPDH antibodies were obtained from Proteintech Group, Inc. The anti-AKT antibody was provided by Affinity Biosciences.

### 2.10. Animal Study

BALB/c nude mice (4-6 weeks old) were provided by Charles River Laboratories, Inc. All animals were maintained following the Guide for the Care and Use of Laboratory Animals by the National Institutes of Health. In addition, the experiments of animal study were approved by the Ethics Committee of HY cell biotechnology (No. HY2021-33). C33A cells at the density of 1 × 10^7^ cells were subcutaneously injected into the left flank of nude mice. Next, when the tumor volume reached ~200 mm^3^, mice were randomly divided into three groups: control, TNF-*α*, and TNF-*α*+MAZ251 groups. TNF-*α* was intraperitoneally injected into mice in TNF-*α* and TNF-*α*+MAZ251 groups three times a week at 54 *μ*g/kg for 3 weeks. In addition, MAZ51 was intraperitoneally injected into mice in the TNF-*α*+MAZ251 group once a day at 8 mg/kg for 15 days. Meanwhile, normal saline was intraperitoneally injected into mice in the control group. The tumor volume was measured weekly according to the following formula: volume = length × width^2^/2 [35]. In 3 weeks, all mice were sacrificed using CO_2_ (40% volume/min). And the tumors were photographed and weighted. Meanwhile, plasma samples were collected using anticoagulation tubes and then centrifuged for 10 min at 2,000 × g at 4°C.

### 2.11. Immunohistochemistry (IHC) Staining

A tumor tissue section was dewaxed with xylene. Antigens from the tumor tissue section were extracted with 0.01 M heated citrate buffers (pH 6.0). Next, 200 *μ*l blocking solution was dropped onto the slices at room temperature for 1 h. The section was then incubated with a primary anti-D2-40 or PDPN antibody overnight at 4°C. Next, the section was washed with PBS for 3 times. Subsequently, the section was incubated with secondary antibodies for 50 min at room temperature. DAB was then used for chromogenic reactions. In addition, the slices were placed in a hematoxylin solution for redyeing. Finally, the coverslip was placed over the section, and the staining results were observed using a microscope (CX31; Olympus Corporation) [36]. ImageJ software (with IHC Profiler plugins) was used for IHC scoring.

### 2.12. Real-Time PCR (RT-PCR)

The TRIpure Total RNA Extraction Reagent (cat. no. EP013; ELK Biotechnology Co., Ltd.) was used to isolate the RNA from cells. Then, the EnTurbo™ SYBR Green PCR SuperMix kit (cat. no. EQ001; ELK Biotechnology Co., Ltd.) was used to perform RT-PCR. The cycling conditions for qPCR were as follows: 95°C for 3 min, followed by 95°C for 10 s, 58°C for 30 s, and 72°C for 30 s for 40 cycles. The information of primers: GAPDH forward, 5′-CATCATCCCTGCCTCTACTGG-3′ and reverse, 5′-GTGGGTGTCGCTGTTGAAGTC-3′; VEGFC forward, 5′-ACGAGCTACCTCAGCAAGACG-3′ and reverse, 5′-CTCCAGCATCCGAGGAAAAC-3′; D2-40 forward, 5′-CTATAAGTCTGGCTTGACAACTCT-3′ and reverse, 5′-CATCTTTCTCAACTGTTGTCTGTG-3′; VEGFR forward, 5′-GGGCATGTACTGACGATTATGG-3′ and reverse, 5′-GGAGGAATGGCATAGACCGTA-3′. The relative level of VEGFC, D2-40, and VEGFR was calculated using the 2^-*ΔΔ*Ct^ method [27, 37].

### 2.13. Statistical Analysis

The statistical analysis was performed using GraphPad Prism software version 7.0 (GraphPad Software, Inc.). Data are presented as the mean ± standard deviation and analyzed using a one-way analysis of variance and Tukey's post hoc test. *P* < 0.05 indicated a statistically significant difference [30, 38].

## 3. Results

### 3.1. TNF-*α* Promotes the Production of VEGFC in Cervical Cancer Cells

**Figure 1:**
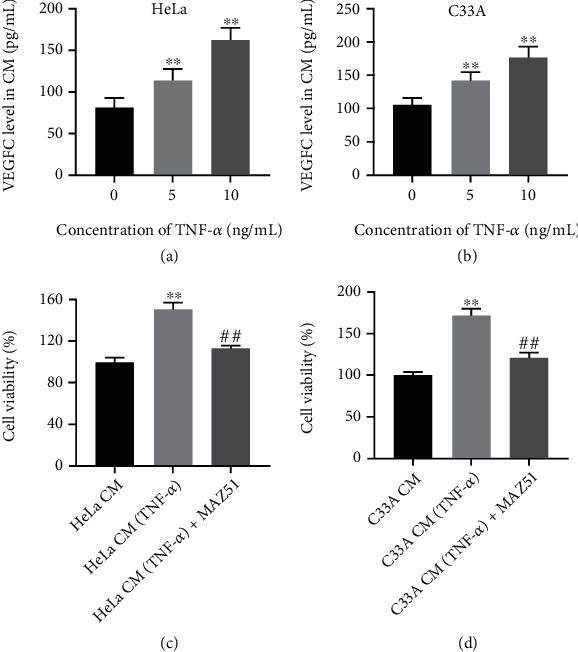
TNF-*α* stimulates cervical cancer cells to secrete VEGFC and promotes HLEC viability. (a, b) Cervical cancer cells (HeLa and C33A) were stimulated with different concentrations of TNF-*α* (0, 5, or 10 ng/ml) for 48 h, and corresponding CM was collected. Next, the level of VEGFC in the corresponding CM was detected by ELISA. (c, d) HLECs were cultured in CM with or without MAZ51 treatment. HLEC viability was measured by CCK-8 assay. ^∗∗^*P* < 0.01 compared with CM group; ^##^*P* < 0.01 compared with CM-TNF-*α* group, *n* = 3.

It has been reported that VEGFC is the most representative and important factor promoting the formation of tumor lymphangiogenesis [39, 40]. In addition, TNF-*α* was found to stimulate cell secretion of VEGF [41]. Our results found that TNF-*α* obviously upregulated the level of VEGFC in the CM of HeLa and C33A cells in a dose-dependent manner (Figures 1(a) and 1(b)). These results showed that TNF-*α* promoted the production of VEGFC in cervical cancer cells.

### 3.2. CM-TNF-*α* Promotes HLEC Viability, Proliferation, and Angiogenesis

**Figure 2:**
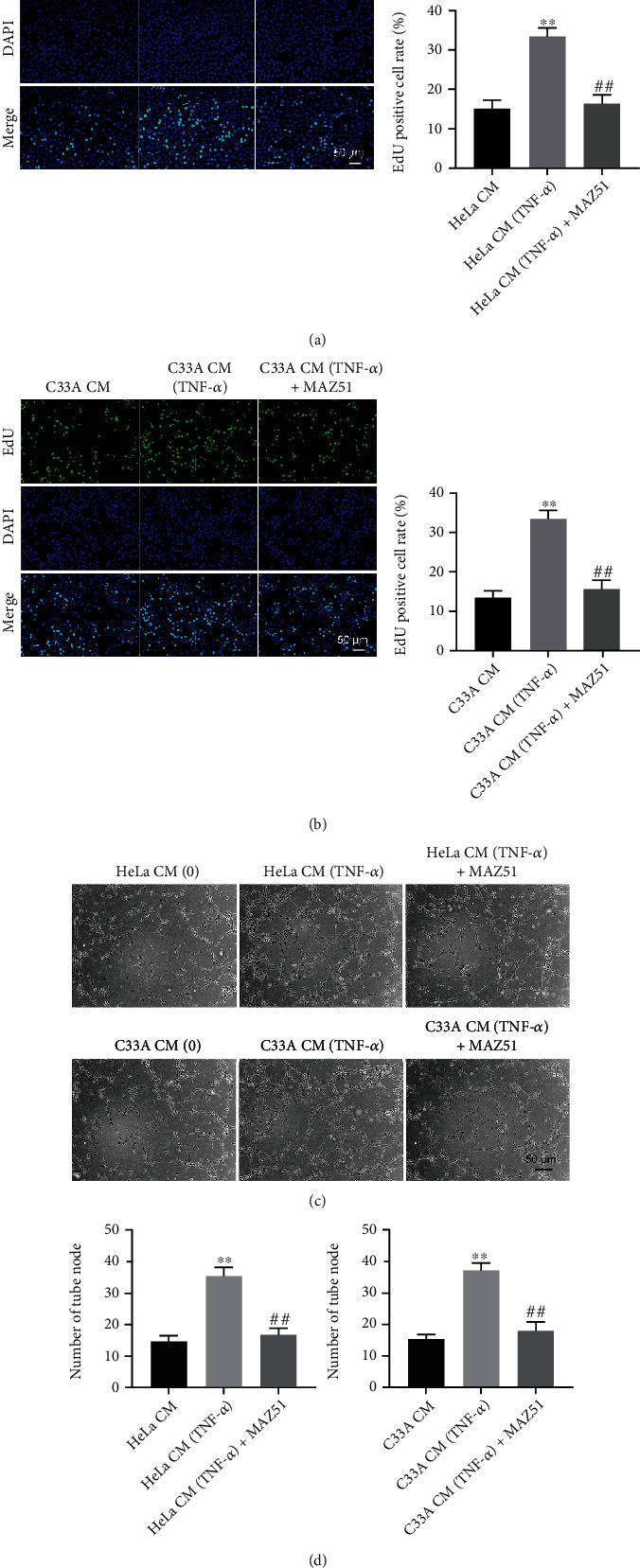
CM-TNF-*α* promotes the proliferation and angiogenesis of HLECs. Cervical cancer cells (HeLa and C33A) were stimulated with 10 ng/ml TNF-*α* for 48 h, and corresponding CM was collected. Next, HLECs were cultured in CM with or without MAZ51 treatment. (a, b) The proliferation of HLECs was measured by EdU staining assay. (c, d) The number of tube node of HLECs was observed using a microscope. ^∗∗^*P* < 0.01 compared with CM group; ^##^*P* < 0.01 compared with CM-TNF-*α* group, *n* = 3.

With the aim of investigating the effect of TNF-*α* on the formation of lymphangiogenesis in cervical cancer, HLECs were cultured in CM-TNF-*α*. As indicated in Figures 1(c) and 1(d) and 2(a) and 2(b), CM-TNF-*α* markedly promoted the viability and proliferation of HLECs. However, these phenomena were reversed in the presence of VEGFR3 inhibitor MAZ51 (Figures 1(c) and 1(d) and 2(a)and 2(b)). Meanwhile, CM-TNF-*α* significantly increased the number of tube node formed in HLECs, and that effect was notably inhibited by MAZ51 (Figures 2(c) and 2(d)). All these results indicated that CM-TNF-*α* could promote HLEC proliferation and angiogenesis by upregulating VEGFC.

### 3.3. CM-TNF-*α* Increases HLEC Migration

**Figure 3:**
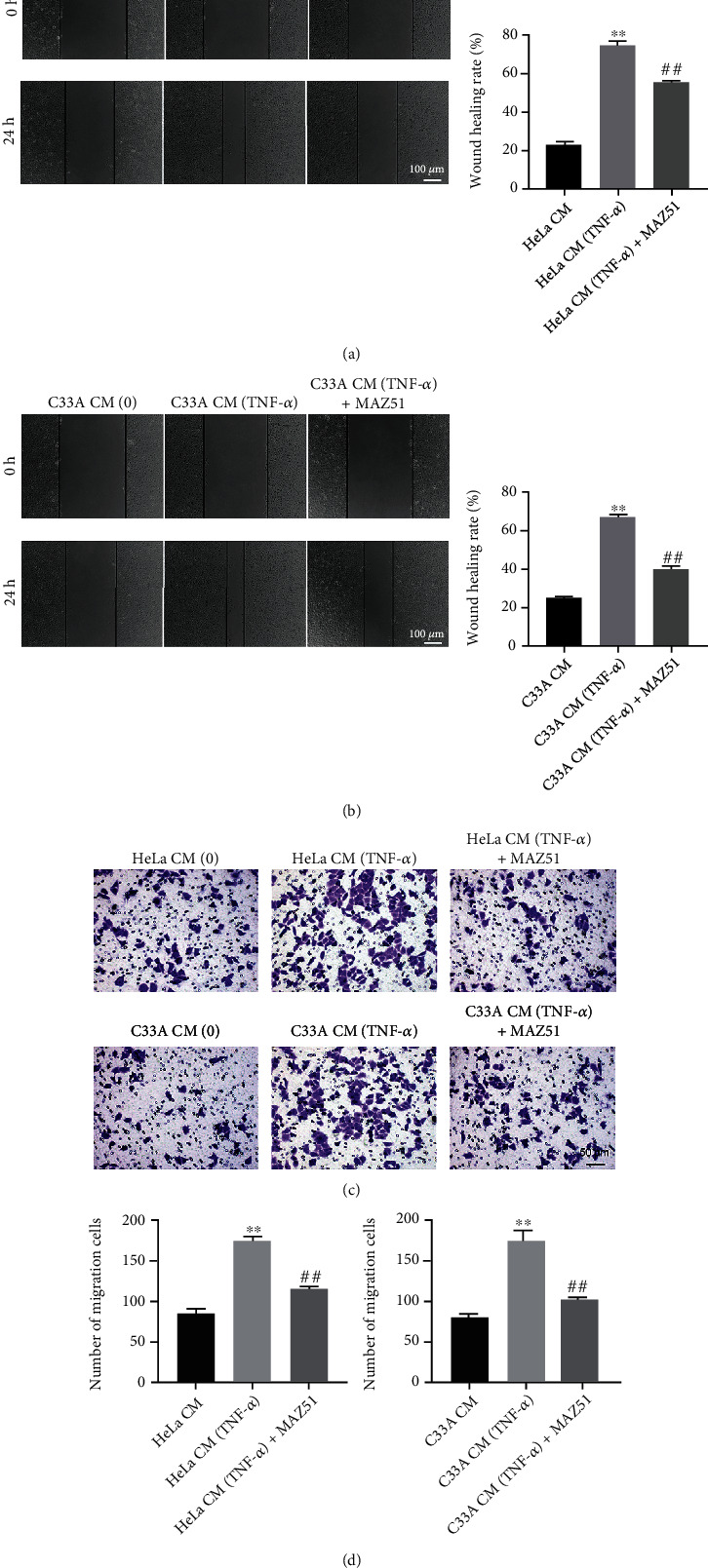
CM-TNF-*α* increases the migration of HLECs. Cervical cancer cells (HeLa and C33A) were stimulated with 10 ng/ml TNF-*α* for 48 h, and corresponding CM was collected. Next, HLECs were cultured in CM with or without MAZ51 treatment. (a, b) The migration of HLECs was measured by wound healing assay. (c, d) The migration of HLECs was measured by transwell migration assay. ^∗∗^*P* < 0.01 compared with CM group; ^##^*P* < 0.01 compared with CM-TNF-*α* group, *n* = 3.

In order to study the role of TNF-*α* on HLEC migration, wound healing, and transwell assays were conducted. The results showed that CM-TNF-*α* significantly promoted HLEC migration, but MAZ51 clearly inhibited this promotion (Figures 3(a)–3(d)). These results showed that CM-TNF-*α* could increase HLEC migration by upregulating VEGFC.

### 3.4. CM-TNF-*α* Upregulates the Expressions of p-AKT and p-ERK of HLECs

**Figure 4:**
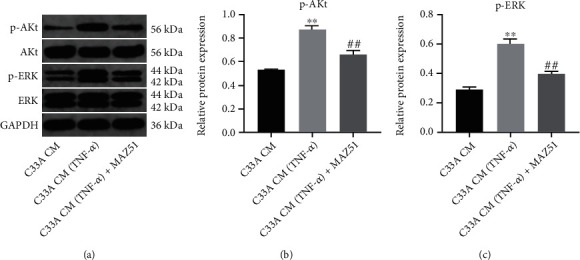
CM-TNF-*α* upregulates the expressions of p-AKT and p-ERK of HLECs. C33A cells were stimulated with 10 ng/ml TNF-*α* for 48 h, and the CM was collected. Next, HLECs were cultured in CM with or without MAZ51 treatment. (a–c) The levels of AKT, p-AKT, ERK, and p-ERK of HLECs were measured by western blot. ^∗∗^*P* < 0.01 compared with CM group; ^##^*P* < 0.01 compared with CM-TNF-*α* group, *n* = 3.

The AKT and ERK signaling pathways have been reported to play an important role in cervical cancer progression [42, 43]. In order to explore the mechanism by which TNF-*α* regulates the lymphangiogenesis of HLECs, the expressions of p-AKT and p-ERK were evaluated by western blotting. The results indicated that CM-TNF-*α* markedly increased the levels of p-AKT and p-ERK in HLECs, and these increases were markedly suppressed by MAZ51 (Figures 4(a)–4(c)). Collectively, CM-TNF-*α* could promote the expressions of p-AKT and p-ERK in HLECs by upregulating VEGFC.

### 3.5. TNF-*α* Promotes the Tumorigenesis, Lymphangiogenesis, and Lymphatic Metastasis of Cervical Cancer *In Vivo*

**Figure 5:**
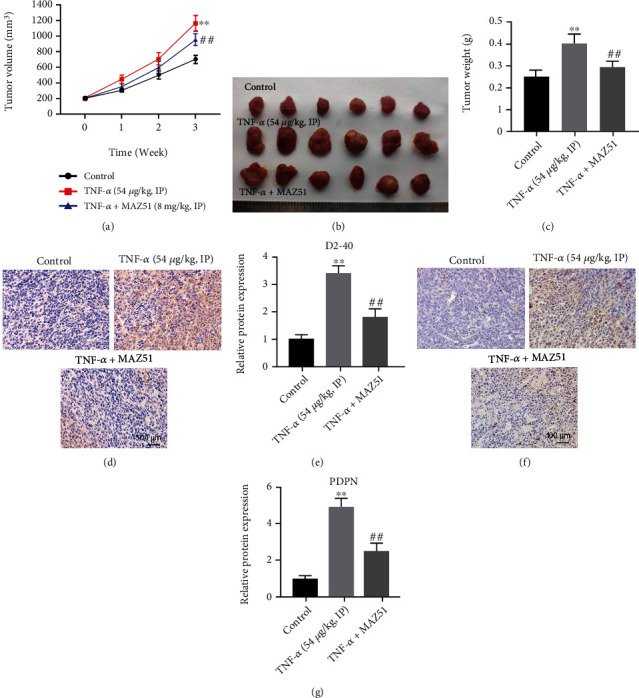
TNF-*α* promotes the tumorigenesis of cervical cancer *in vivo*. (a) The tumor volume was measured weekly. (b, c) The tumors were photographed and weighted. (d–g) The level of D2-40 or PDPN in tumor tissue was measured by IHC staining assay. ^∗∗^*P* < 0.01 compared with control group; ^##^*P* < 0.01 compared with TNF-*α* treated group, *n* = 3.

Finally, to confirm the effect of TNF-*α* on cervical cancer tumorigenesis and lymph node metastasis, a C33A subcutaneous xenograft model was established *in vivo*. As shown in Figures 5(a)–5(c), TNF-*α* remarkably promoted tumor volume and weight in C33A subcutaneous xenografts; however, this promotion was clearly inhibited by MAZ51 treatment. In addition, D2-40 has been reported as a specific marker of lymphatic endothelial cells that can be used in the study of lymph node metastasis [44, 45]. The IHC results suggested that TNF-*α* visibly increased D2-40 and PDPN levels in tumor tissues, and these increases were reversed by MAZ51 (Figures 5(d)–5(g)).

**Figure 6:**
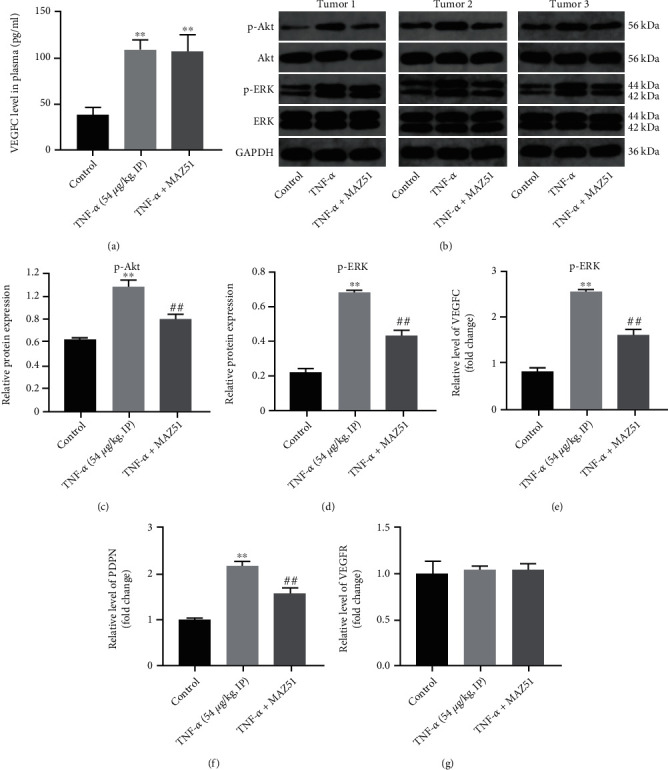
TNF-*α* upregulates p-AKT and p-ERK expression in tumor tissues via mediating VEGFC signaling *in vivo*. (a) The level of VEGFC in the plasma was measured by ELISA. (b–d) The levels of AKT, p-AKT, ERK, and p-ERK in tumor tissue were measured by western blot. (e–g) The levels of VEGFC, D2-40, and VEGFR were detected with RT-qPCR. ^∗∗^*P* < 0.01 compared with control group; ^##^*P* < 0.01 compared with TNF-*α* treated group, *n* = 3.

Furthermore, ELISA results suggested that TNF-*α* significantly increased VEGFC expression in the plasma *in vivo*, which was reversed by MAZ51 (Figure 6(a)). Meanwhile, TNF-*α* visibly increased p-AKT and p-ERK expressions in tumor tissues, but these increases were reversed by MAZ51 (Figures 6(b)–6(d)). In addition, TNF-*α* upregulated the levels of VEGFC and D2-40 in tumor tissues compared with the control group (Figures 6(e) and 6(f)). Meanwhile, compared with the control group, TNF-*α* had few effects on VEGFR gene expression in tumor tissues (Figure 6(g)). In general, TNF-*α* could promote cervical cancer tumorigenesis, lymphangiogenesis, and lymphatic metastasis *in vivo* via activating VEGFC-mediated AKT and ERK pathways.

## 4. Discussion

TNF-*α* is an important regulator of the inflammatory response [46, 47] that can activate neutrophils and lymphocytes, increase vascular endothelial cell permeability, and regulate cellular and tissues metabolism [46, 48]. Therefore, studying the mechanism of inflammatory factors to promote tumor progression and metastasis has become a research hotspot in recent years. For instance, TNF-*α* has been reported to promote tumor development and metastasis [24, 49]. Forkasiewicz et al. found that TNF-*α* could promote esophageal cancer cell migration [50]. In addition, Liang et al. showed that TNF-*α* could enhance gastric cancer cell migration and invasion via activating NF-*κ*B signaling [51]. Consistent with that study, the present data indicated that CM-TNF-*α* could promote the growth of cervical cancer cells *in vivo*. Furthermore, this study showed that TNF-*α* could stimulate cervical cancer cells to secrete VEGFC, which in turn promoted the proliferation, migration, and angiogenesis of HLECs. Therefore, this study was the first to explore the effect of TNF-*α* on the progression of cervical cancer from the perspective of the TME.

The interaction between cancer cells and lymphatic endothelial cells is crucial in promoting tumor growth and metastasis in the TME, as demonstrated by previous studies [52, 53]. One of these studies showed that lymphatic endothelial cells could enhance the proliferation and migration of tumor cells [54], while cancer cells could also accelerate endothelial cell tube formation via activating the PI3K/Akt pathway [55]. Moreover, tumor-induced lymphangiogenesis is known to play a vital role in the initial stages of cancer metastasis [56], andthe link between VEGFC and tumor lymphangiogenesis and metastasis has been extensively investigated [57, 58]. Chen et al. found that cancer cell-derived VEGFC could promote lymphangiogenesis in lymph nodes, which in return promotes cancer metastasis [59]. Meanwhile, He et al. showed that VEGFC could promote cervical cancer metastasis [60]. Besides, researchers have found that TNF-*α* could upregulate VEGFC expression, promoting lymphangiogenesis and lymphatic metastasis in gallbladder cancer [61]. In our study, we observed that the expression of VEGFC was significantly increased in the CM collected from the TNF-*α*-treated cervical cancer cells. CM collected from these cells also promoted the proliferation, migration, and angiogenesis of HLECs; and these changes were reversed by MAZ51, a VEGFR3 inhibitor. Furthermore, TNF-*α*elevated D2-40 and VEGFC protein expressions in tumor tissues, indicating that TNF-*α* could promote lymphangiogenesis and lymphatic metastasis of cervical cancer *in vivo*. Our findings suggest that TNF-*α* could be apromising target for cervical cancer treatment, as it promote lymphangiogenesis and lymphatic metastasis by upregulating VEGFC.

TNF-*α* was found to induce colorectal cancer cell migration and EMT via activating AKT signaling [62]. In addition, TNF-*α* could promote triple-negative breast cancer cell metastasis through targeting TNFR2-ERK1/2-EZH2 signaling [63]. These findings showed that TNF-*α* could promote tumor development via modulating AKT and ERK signaling pathways. Additionally, the literature suggested that the AKT and ERK signaling pathways are extensively involved in cervical cancer development [64–66]. For example, exosomal miR-221-3p secreted by cervical squamous cell carcinoma promoted the formation and metastasis of HLECs by upregulating the AKT/ERK pathway [67]. In addition, protein tyrosine phosphatase receptor M can induce lymphangiogenesis and lymph node metastasis through the AKT signaling pathway in a VEGFC-dependent manner [68]. In the present study, the CM collected from the TNF-*α*-treated cervical cancer cells was found to increase p-AKT and p-ERK expressions in HLECs. Moreover, TNF-*α* could upregulate p-AKT and p-ERK expressions in tumor tissues. However, inhibition of VEGFR3 obviously reversed these changes. All these data suggested that the AKT and ERK signaling pathways are involved in the lymphangiogenesis in cervical cancer.

In this study, we only determined that TNF-*α* could inhibit cervical cancer progression by targeting VEGFC-mediated AKT and ERK pathways. Thus, further study is needed to investigate whether TNF-*α* could affect the progression of cervical cancer via targeting other pathways, such as AMPK/mTOR or the NF-*κ*B signaling pathway [69, 70].

## 5. Conclusion

To sum up, our studyrevealed that TNF-*α*activatesVEGFC-mediated AKT and ERK pathways, leading to tumorigenesis, lymphangiogenesis, and lymphatic metastasis *in vitro* and *in vivo* in cervical cancer. Wehope that our research will provide new directions for the treatment of cervical cancer.

## Data Availability

All data, models, and code generated or used during the study are available from the corresponding author upon reasonable request.

## References

[1] Vu M., Yu J., Awolude O. A., Chuang L. (2018). Cervical cancer worldwide. *Current Problems in Cancer*.

[2] Tsikouras P., Zervoudis S., Manav B. (2016). Cervical cancer: screening, diagnosis and staging. *Journal of BUON: Official Journal of the Balkan Union of Oncology*.

[3] Lin W., Feng M., Chen G., Zhou Z., Li J., Ye Y. (2017). Characterization of the microRNA profile in early-stage cervical squamous cell carcinoma by next-generation sequencing. *Oncology Reports*.

[4] Mei J., Zhu C., Pan L., Li M. (2022). MACC1 regulates the AKT/STAT3 signaling pathway to induce migration, invasion, cancer stemness, and suppress apoptosis in cervical cancer cells. *Bioengineered*.

[5] Olusola P., Banerjee H. N., Philley J. V., Dasgupta S. (2019). Human papilloma virus-associated cervical cancer and health disparities. *Cell*.

[6] Lin W., Feng M., Li X. (2017). Transcriptome profiling of cancer and normal tissues from cervical squamous cancer patients by deep sequencing. *Molecular Medicine Reports*.

[7] Zhong Y., Lu Q., Qiu W., Luo Y. (2020). LINC00636 promotes lymph node metastasis and cervical cancer through targeting NM23. *Bioscience Reports*.

[8] Huang C., Chen Y. (2017). Lymphangiogenesis and colorectal cancer. *Saudi Medical Journal*.

[9] Cohen P., Jhingran A., Oaknin A., Denny L. (2019). Cervical cancer. *The Lancet*.

[10] Vordermark D. (2016). Radiotherapy of cervical cancer. *Oncology Research and Treatment*.

[11] Kumar L., Harish P., Malik P. S., Khurana S. (2018). Chemotherapy and targeted therapy in the management of cervical cancer. *Current Problems in Cancer*.

[12] Marquina G., Manzano A., Casado A. (2018). Targeted agents in cervical cancer: beyond bevacizumab. *Current Oncology Reports*.

[13] Hinshaw D. C., Shevde L. A. (2019). The tumor microenvironment innately modulates cancer progression. *Cancer Research*.

[14] Iyengar N. M., Gucalp A., Dannenberg A. J., Hudis C. A. (2016). Obesity and cancer mechanisms: tumor microenvironment and inflammation. *Journal of Clinical Oncology: Official Journal of the American Society of Clinical Oncology*.

[15] Rizzo A., Pallone F., Monteleone G., Fantini M. C. (2011). Intestinal inflammation and colorectal cancer: a double-edged sword?. *World Journal of Gastroenterology*.

[16] Chaudhry H., Zhou J., Zhong Y. (2013). Role of cytokines as a double-edged sword in sepsis. *In Vivo (Athens, Greece)*.

[17] Schreiner P., Biedermann L. (2019). Editorial: anti-TNF therapy—a double-edged sword?. *Alimentary Pharmacology & Therapeutics*.

[18] Garancher A., Suzuki H., Haricharan S. (2020). Tumor necrosis factor overcomes immune evasion in p53-mutant medulloblastoma. *Nature Neuroscience*.

[19] Wang T., Chen B., Meng T., Liu Z., Wu W. (2021). Identification and immunoprofiling of key prognostic genes in the tumor microenvironment of hepatocellular carcinoma. *Bioengineered*.

[20] Lin E. W., Karakasheva T. A., Hicks P. D., Bass A. J., Rustgi A. K. (2016). The tumor microenvironment in esophageal cancer. *Oncogene*.

[21] Patel H. J., Patel B. M. (2017). TNF-*α* and cancer cachexia: molecular insights and clinical implications. *Life Sciences*.

[22] Coppack S. W. (2001). Pro-inflammatory cytokines and adipose tissue. *The Proceedings of the Nutrition Society*.

[23] Tamburrini E., De Luca A., Ventura G. (1990). Macrophages from healthy adults release TNF-alpha after exposure to Pneumocystis carinii of murine origin. Preliminary study. *Medicina (Florence, Italy)*.

[24] Cruceriu D., Baldasici O., Balacescu O., Berindan-Neagoe I. (2020). The dual role of tumor necrosis factor-alpha (TNF-*α*) in breast cancer: molecular insights and therapeutic approaches. *Cellular Oncology (Dordrecht)*.

[25] Yoshimatsu Y., Wakabayashi I., Kimuro S. (2020). TNF-*α* enhances TGF-*β*-induced endothelial-to-mesenchymal transition via TGF-*β* signal augmentation. *Cancer Science*.

[26] Fujiki H., Suganuma M. (2011). Tumor promoters--microcystin-LR, nodularin and TNF-*α* and human cancer development. *Anti-Cancer Agents in Medicinal Chemistry*.

[27] Chen C., Luo Y., He W. (2020). Exosomal long noncoding RNA LNMAT2 promotes lymphatic metastasis in bladder cancer. *The Journal of Clinical Investigation*.

[28] Li Q., Liu L., Zhang Q., Liu S., Ge D., You Z. (2014). Interleukin-17 indirectly promotes M2 macrophage differentiation through stimulation of COX-2/PGE2 pathway in the cancer cells. *Cancer Research and Treatment*.

[29] Yu J., Li Y., Li Z. (2021). Subconjunctival injections of dimethyl fumarate inhibit lymphangiogenesis and allograft rejection in the rat cornea. *International Immunopharmacology*.

[30] Wang X., Sun H., Zhu S. (2021). Long non-coding RNA PTAR inhibits apoptosis but promotes proliferation, invasion and migration of cervical cancer cells by binding miR-101. *Bioengineered*.

[31] Shi G., Yang F. (2021). Krüppel-like factor 1 (KLF1) promoted the proliferation, migration and invasion of human lens epithelial cells by enhancing the expression of zinc finger and BTB domain containing 7A (ZBTB7A) and activating Wnt/*β*-catenin pathway. *Bioengineered*.

[32] Han H., Xu X. (2022). MiR-205 promotes the viability, migration, and tube formation of cervical cancer cells *in vitro* by targeting *GATA3*. *Cancer Biotherapy & Radiopharmaceuticals*.

[33] Yang Y., Xiao C., Liu K., Song L., Zhang Y., Dong B. (2021). Silencing of long noncoding INHBA antisense RNA1 suppresses proliferation, migration, and extracellular matrix deposition in human hypertrophic scar fibroblasts via regulating microRNA-141-3p/myeloid cell leukemia 1 axis. *Bioengineered*.

[34] He X., Li C., Yin H. (2022). Mesenchymal stem cells inhibited the apoptosis of alveolar epithelial cells caused by ARDS through CXCL12/CXCR4 axis. *Bioengineered*.

[35] Zhu S. Y., Wu Q. Y., Zhang C. X. (2018). miR-20a inhibits the killing effect of natural killer cells to cervical cancer cells by downregulating RUNX1. *Biochemical and Biophysical Research Communications*.

[36] Zan L., Chen Q., Zhang L., Li X. (2019). Epigallocatechin gallate (EGCG) suppresses growth and tumorigenicity in breast cancer cells by downregulation of miR-25. *Bioengineered*.

[37] Ni M., Yan Q., Xue H., Du Y., Zhao S., Zhao Z. (2021). Identification of MYLIP gene and miRNA-802 involved in the growth and metastasis of cervical cancer cells. *Cancer Biomarkers: Section A of Disease Markers*.

[38] Shou Y., Wang X., Liang Y., Liu X., Chen K. (2022). Exosomes-derived miR-154-5p attenuates esophageal squamous cell carcinoma progression and angiogenesis by targeting kinesin family member 14. *Bioengineered*.

[39] Raica M., Cimpean A. M., Ceausu R., Ribatti D. (2011). Lymphatic microvessel density, VEGF-C, and VEGFR-3 expression in different molecular types of breast cancer. *Anticancer Research*.

[40] Franc M., Kachel-Flis A., Michalski B. (2015). Lymphangiogenesis in cervical cancer evaluated by expression of the *VEGF-C* gene in clinical stage IB-IIIB. *Menopause Review/Przegląd Menopauzalny*.

[41] Wang M., Crisostomo P. R., Herring C., Meldrum K. K., Meldrum D. R. (2006). Human progenitor cells from bone marrow or adipose tissue produce VEGF, HGF, and IGF-I in response to TNF by a p38 MAPK-dependent mechanism. *American Journal of Physiology. Regulatory, Integrative and Comparative Physiology*.

[42] Zhang W., Zhou Q., Wei Y. (2019). The exosome-mediated PI3k/Akt/mTOR signaling pathway in cervical cancer. *International Journal of Clinical and Experimental Pathology*.

[43] Ma H., Han F., Yan X. (2021). PBK promotes aggressive phenotypes of cervical cancer through ERK/c-Myc signaling pathway. *Journal of Cellular Physiology*.

[44] Abe N., Ohtake T., Saito K., Kumamoto K., Sugino T., Takenoshita S. (2016). Clinicopathological significance of lymphangiogenesis detected by immunohistochemistry using D2-40 monoclonal antibody in breast cancer. *Fukushima Journal of Medical Science*.

[45] Braun M., Flucke U., Debald M. (2008). Detection of lymphovascular invasion in early breast cancer by D2-40 (podoplanin): a clinically useful predictor for axillary lymph node metastases. *Breast Cancer Research and Treatment*.

[46] Wen Y., Rudemiller N. P., Zhang J. (2020). TNF-*α* in T lymphocytes attenuates renal injury and fibrosis during nephrotoxic nephritis. *American Journal of Physiology Renal Physiology*.

[47] Yan F., Wufuer D., Ding J., Wang J. (2021). MicroRNA miR-146a-5p inhibits the inflammatory response and injury of airway epithelial cells via targeting TNF receptor-associated factor 6. *Bioengineered*.

[48] Lu Y., Lian Z., Yang H. (2020). TNF-*α* activates RhoA/ROCK signaling pathway and increases permeability of endothelial cells infected with Listeria monocytogenes. *Xi Bao yu fen zi Mian yi xue za zhi = Chinese Journal of Cellular and Molecular Immunology*.

[49] Hu C. W., Chang Y. C., Liu C. H., Yu Y. A., Mou K. Y. (2022). Development of a TNF-*α*-mediated Trojan horse for bacteria-based cancer therapy. *Molecular Therapy: The Journal of the American Society of Gene Therapy*.

[50] Forkasiewicz A., Stach W., Wierzbicki J. (2022). Effect of LDHA inhibition on TNF-*α*-induced cell migration in esophageal cancers. *International Journal of Molecular Sciences*.

[51] Liang X., Feng Z., Yan R. (2022). Kruppel-like factors 3 regulates migration and invasion of gastric cancer cells through NF-*κ*B pathway. *Alternative Therapies in Health and Medicine*.

[52] Lee E., Pandey N. B., Popel A. S. (2015). Crosstalk between cancer cells and blood endothelial and lymphatic endothelial cells in tumour and organ microenvironment. *Expert Reviews in Molecular Medicine*.

[53] Lee B. S., Jang J. Y., Seo C., Kim C. H. (2021). Crosstalk between head and neck cancer cells and lymphatic endothelial cells promotes tumor metastasis via CXCL5-CXCR2 signaling. *FASEB Journal: Official Publication of the Federation of American Societies for Experimental Biology*.

[54] Van de Velde M., Ebroin M., Durré T. (2021). Tumor exposed-lymphatic endothelial cells promote primary tumor growth via IL6. *Cancer Letters*.

[55] Cheng H. W., Chen Y. F., Wong J. M. (2017). Cancer cells increase endothelial cell tube formation and survival by activating the PI3K/Akt signalling pathway. *Journal of Experimental & Clinical Cancer Research : CR*.

[56] Li S., Li Q. (2015). Cancer stem cells, lymphangiogenesis, and lymphatic metastasis. *Cancer Letters*.

[57] Lin W., Jiang L., Chen Y. (2012). Vascular endothelial growth factor-D promotes growth, lymphangiogenesis and lymphatic metastasis in gallbladder cancer. *Cancer Letters*.

[58] Ji R. C. (2006). Lymphatic endothelial cells, tumor lymphangiogenesis and metastasis: new insights into intratumoral and peritumoral lymphatics. *Cancer Metastasis Reviews*.

[59] Chen J. Y., Lai Y. S., Chu P. Y., Chan S. H., Wang L. H., Hung W. C. (2019). Cancer-derived VEGF-C increases chemokine production in lymphatic endothelial cells to promote CXCR2-dependent cancer invasion and MDSC recruitment. *Cancers*.

[60] He M., Cheng Y., Li W. (2010). Vascular endothelial growth factor C promotes cervical cancer metastasis via up-regulation and activation of RhoA/ROCK-2/moesin cascade. *BMC Cancer*.

[61] Li C. Z., Jiang X. J., Lin B. (2018). RIP1 regulates TNF-*α*-mediated lymphangiogenesis and lymphatic metastasis in gallbladder cancer by modulating the NF-*κ*B-VEGF-C pathway. *OncoTargets and Therapy*.

[62] Wang H., Wang H. S., Zhou B. H. (2013). Epithelial-mesenchymal transition (EMT) induced by TNF-*α* requires AKT/GSK-3*β*-mediated stabilization of snail in colorectal cancer. *PLoS One*.

[63] Mei X. Y., Zhang J. N., Jia W. Y. (2022). Scutellarin suppresses triple-negative breast cancer metastasis by inhibiting TNF*α*-induced vascular endothelial barrier breakdown. *Acta Pharmacologica Sinica*.

[64] Chen Y. H., Yang S. F., Yang C. K. (2020). Metformin induces apoptosis and inhibits migration by activating the AMPK/p53 axis and suppressing PI3K/AKT signaling in human cervical cancer cells. *Molecular Medicine Reports*.

[65] Che Y., Li Y., Zheng F. (2019). TRIP4 promotes tumor growth and metastasis and regulates radiosensitivity of cervical cancer by activating MAPK, PI3K/AKT, and hTERT signaling. *Cancer Letters*.

[66] Lu X., Song X., Hao X. (2021). miR-186-3p attenuates the tumorigenesis of cervical cancer via targeting insulin-like growth factor 1 to suppress PI3K-Akt signaling pathway. *Bioengineered*.

[67] Zhou C. F., Ma J., Huang L. (2019). Cervical squamous cell carcinoma-secreted exosomal miR-221-3p promotes lymphangiogenesis and lymphatic metastasis by targeting VASH1. *Oncogene*.

[68] Liu P., Zhang C., Liao Y. (2020). High expression of PTPRM predicts poor prognosis and promotes tumor growth and lymph node metastasis in cervical cancer. *Cell Death & Disease*.

[69] Zhao X., Lin Y., Jiang B. (2020). Icaritin inhibits lung cancer-induced osteoclastogenesis by suppressing the expression of IL-6 and TNF-a and through AMPK/mTOR signaling pathway. *Anti-Cancer Drugs*.

[70] Mukhopadhyay A., Shishodia S., Suttles J. (2002). Ectopic expression of protein-tyrosine kinase Bcr-Abl suppresses tumor necrosis factor (TNF)-induced NF-*κ*B activation and I*κ*B*α* phosphorylation. *The Journal of Biological Chemistry*.

